# College and Hospital Notes

**Published:** 1903-01

**Authors:** 


### COLLEGE AND HOSPITAL NOTES.

Dr. Lillian Craig Randall, of Riverside Hospital, is establishing an accident hospital in the Fitch Institute Building, Michigan and Swan Streets, to be known as the Riverside Accident Hospital. For many years there were two accident hospitals in that section of the city, but the Fitch Hospital was closed and the Emergency Hospital removed to Pine and Eagle Streets, leaving the old neighborhood without an institution of the kind.

A relief station, known as first relief station of the Riverside Accident Hospital, has been opened in a cottage at No. 796 Tonawanda Street. Four rooms have been fitted up for the first care of the injured, the Riverside Hospital ambulance serving in emergency calls. The relief station will be used only for giving the first dressing and if the patient needs further hospital care, he will be removed to the hospital in Lafayette Avenue. A trained nurse will be in attendance throughout the day and Dr. J. H. Potter and Dr. John C. Thompson, who are in the vicinity, will give medical attendance. Owing to the numerous accidents in the factories and other establishments in Black Rock, the relief station is greatly needed.

---

At New Haven, Conn., the tuberculosis hospital of which there has been talk of building for some years is to be built within a short time, recent gifts of land on Woodbridge Heights having assured the success of the undertaking. The hospital will be run mainly on the fresh air theory.

---

An Infant incubator outfit has been installed in the Children's Hospital, 219 Bryant Street, Buffalo, N. Y. The board of managers desire to acquaint physicians and all interested in this city and the surrounding country with this fact, and that they are in readiness to care for any infant requiring such treatment. They also wish to state that the hospital is now prepared to receive contagious cases occurring in children under fourteen years of age.

---

The governors of the New York Skin and Cancer Hospital announce that Dr. L. Duncan Bulkley will give a fifth series of clinical lectures on diseases of the skin in the out-patient hall of the hospital on Wednesday afternoons, commencing January 7, 1903, at 4.15 o'clock. The course will be free to the medical profession.

---

Batavia is about to build a smallpox hospital, plans having been submitted by Architect Hyde and accepted by the town and village boards of health. Work will be commenced soon on the building, which will be erected on River Street in that village.

